# Analysis on the Composition/structure and Lacquering Techniques of the Coffin of Emperor Qianlong Excavated from the Eastern Imperial Tombs

**DOI:** 10.1038/s41598-017-08933-8

**Published:** 2017-08-24

**Authors:** Xinying Hao, Hao Wu, Yang Zhao, Tong Tong, Xiaoyuan Li, Cui Yang, Yun Tang, Xinyu Shen, Hua Tong

**Affiliations:** 1 Key Laboratory of Analytical Chemistry for Biology and Medicine, Ministry of Education, College of Chemistry and Molecular Sciences, Wuhan University, Wuhan, 430072 China; 2 Jingzhou Preservation Centre of Cultural Relics, Jingzhou, 434020 China; 3 Centre of Cultural Material Conservation, The University of Melbourne, Parkville, VIC 3010 Australia; 4 Archaeology Research Center of Science and Technology, Wuhan University, Wuhan, 430072 China

## Abstract

This article presents the results of an investigation on the coffin of Emperor Qianlong excavated from the Eastern Imperial Tombs of the Qing Dynasty in Zunhua, China. The composition, structure and lacquering techniques used in the manufacturing process were analyzed in this project. Stereoscopic Microscopy, SEM-EDS, XRD, FTIR, Raman, Double-shot Py-GC/MS were used as scientific analytical methods. The results show that the structure of the coffin body consists of a wooden body layer, a lacquer ash layer and a lacquer film pigment layer. The lacquer ash layer consists of nine stucco layers and ten fiber layers on top of each other in an alternating order. The lacquer film pigment layer consists of mineral pigments, lacquer sap, animal gelatin, drying oil, quartz sand and proteinaceous materials. Pigments used in the lacquer film include calcite white, carbon black, cinnabar red and gold. The presence of three distinctive catechols along with the other catechols’ and phenols’ profiles in the lacquer film matrix clearly indicate the species of the lacquer tree was Rhus. Vernicifera. Several distinctive lacquering techniques that improved the coffin body’s stability and mechanical strength were identified in the investigation, including the “wan lacquering”, “painting lacquer above the gold” and “Jin Jiao”.

## Introduction

The lacquer craftsmanship in China has a long history that can be traced back to the Neolithic Age. Since the sap of lac trees gives excellent durability, brilliance and longer preservation time to lacquered wares, it has been highly appreciated as a coating material and an adhesive in traditional artistic craftsmanship. Oriental lacquer is categorized into three types based on the original regions of the lacquer trees - Rhus vernicifera (the phenol derivative is urushiol), Rhus succedanea (the phenol derivative is laccol), and Melanorrhoea usitate (the phenol derivative is thitsiol)^1^. These monomers are considered as the most characteristic markers to identify particular species of lacquer^2^. Lacquered wooden coffins are a significant part of the funerary culture in the ancient China and have appeared since the Warring States Period^3^. Imperial coffins were used to store the remains of emperors and empresses. They also often contain mysterious and secretive burial treasures, thus raise great attentions among archaeologists, scholars and the general public.

Eastern Imperial Tombs of the Qing Dynasty are located northwest of the Zunhua County, Tangshan City, Hebei Province of China. The size of the entire mausoleum is 80 square kilometers, making it the largest imperial mausoleum architecture group that now exists in China. Emperor Qianlong was the sixth emperor of the Qing Dynasty (1711–1799 AD). He had the longest consecutive reign and lifespan of all emperors in Chinese history. During his reign, the unification of the multi-ethnic country was accomplished, and the culture, economy, and handicraft industry developed greatly. China reached the highest peak of ‘the Prosperity Epoch of Kangxi and Qianlong’ under his reign. The imperial coffins of Emperor Qianlong and his empresses hold especially significant historic and artistic values, making this project necessary and much desired from the perspectives of both the scholars and the wider public. Upon initial examination on site, the coffin of Emperor Qianlong is observed to be undergoing considerable deterioration from long-term underground storage. The surface decoration has begun to lose its gloss and crack, and will soon start to flake off significantly without proper management of its preservation conditions or taking conservation measures^4^. Therefore, it is vital to develop a technical study methodology for the investigation on the composition of the coffin and the lacquering techniques, in order to gain knowledge on the materials and techniques employed in its manufacture, and to assess the deterioration conditions of the coffins^4^ for future conservation plans.

Instrumental analysis methods such as Stereoscopic Microscopy^5, 6^, Scanning Electron Microscopy by X-Ray Energy Dispersive Spectroscopy (SEM-EDS)^7–9^, X-Ray Diffraction analysis (XRD), Raman Spectroscopy analysis^10–14^, Fourier-transform Infrared Spectroscopy (FTIR)^15–17^, and Pyrolysis-Gas Chromatography and Mass Spectrometry (py-GC-MS)^18–22^ are now widely used by fellow researchers in the technical studies of archaeological artifacts. However, previous studies that employed these technical research tools often focused only on the materials of the artifacts, and rarely mention the craftsmanship, nor do they make a clear connection between the manufacturing techniques and the chemical analysis results. The history of manufacturing lacquerware went through different stages during its development, thus the materials, composition, structure and lacquering craftsmanship of lacquerware from different historic periods were also varied. The differences between lacquerware are not only reflected in the different materials that were used, but rather in the methods of combining the additives into the production of the lacquer, as well as the lacquering techniques when applying onto the surface of the objects. Imperial lacquerware is the essence of Chinese lacquerware, representing the highest level of lacquerware manufacturing technology. The materials and lacquering craftsmanship were more complicated and distinctive. The mutual interference between the complex materials and the lacquering craftsmanship makes this investigation more challenging, interesting, and valuable.

The aim of this project is to achieve a thorough understanding of the materials, composition, structure and lacquering techniques of the lacquered wooden coffin of Emperor Qianlong through various analytical methods. Each analytical method is carefully selected and analytical tests designed so that each sets of analytical results would be able to complement and verify each other. These results revealed useful information on the structure and chemical state of the stucco, the fibers, the pigments, the species of lacquer and the additives. Such information provides crucial facts and evidence for fellow researchers and conservators in the field to understand the composition of the original materials, and how imperial lacquerware were manufactured in this time, as well as for them to obtain historic, artistic and technical interpretations of the Qing Dynasty period. As a result of the investigation, Stereoscopic Microscopy, SEM-EDS and XRD were used for the characterization of the surface lacquer film pigment layer, the lacquer ash layer and the wooden body layer of the coffin. Additional FTIR, Raman and Double-shot Py-GC/MS analysis were performed to further analyze the detailed chemical composition of the lacquer film matrix, and to identify the species of the lacquer used in the manufacturing of this lacquered wooden coffin.

## Results and Discussion

### The Surface Morphology and Composition Analysis

**Figure 1:**
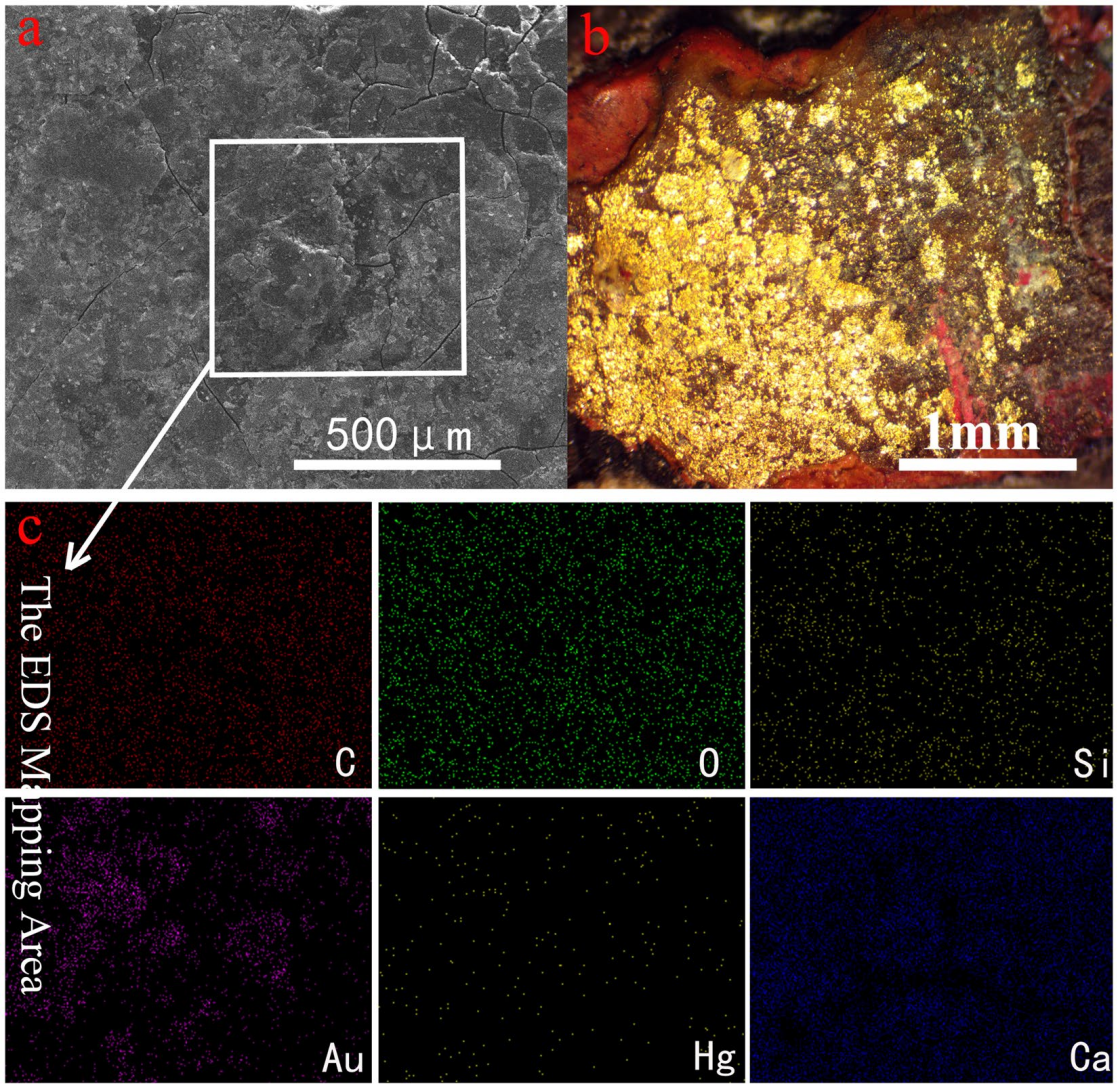
The surface images of the sample from the lacquered wooden coffin of Emperor Qianlong: (**a**) SEM image, (**b**) Stereo Microscope photograph, (**c**) EDS mapping images.

Surface morphology analysis of the samples from the lacquered wood from the coffin was the core step of this project (Fig. 1). Figure 1a shows the SEM image of the surface of a sample from the lacquered wooden coffin, and Fig. 1b shows the Stereo Microscope photograph of the surface of the same sample. Although the sample remained relatively stable as dense layers, some cracking, warping and corrosion pits were still observed on the surface. The cracks have likely led to the flaking of the pigment layer, indicating that the coffin is undergoing considerable deterioration, and effective conservation measures should be taken as soon as possible. In addition, SEM Elemental Mapping analysis was conducted to obtain detailed elemental distribution information (Fig. 1c). As expected, carbon, oxygen, silicon, mercury and calcium atoms are homogeneously distributed throughout the lacquer film pigment layer, reflecting the sophisticated lacquering technology performed on this piece. However, the distribution of gold atoms is heterogeneous, indicating that the gilding is greatly impacted by the long-term deterioration of the coffin.

**Figure 2:**
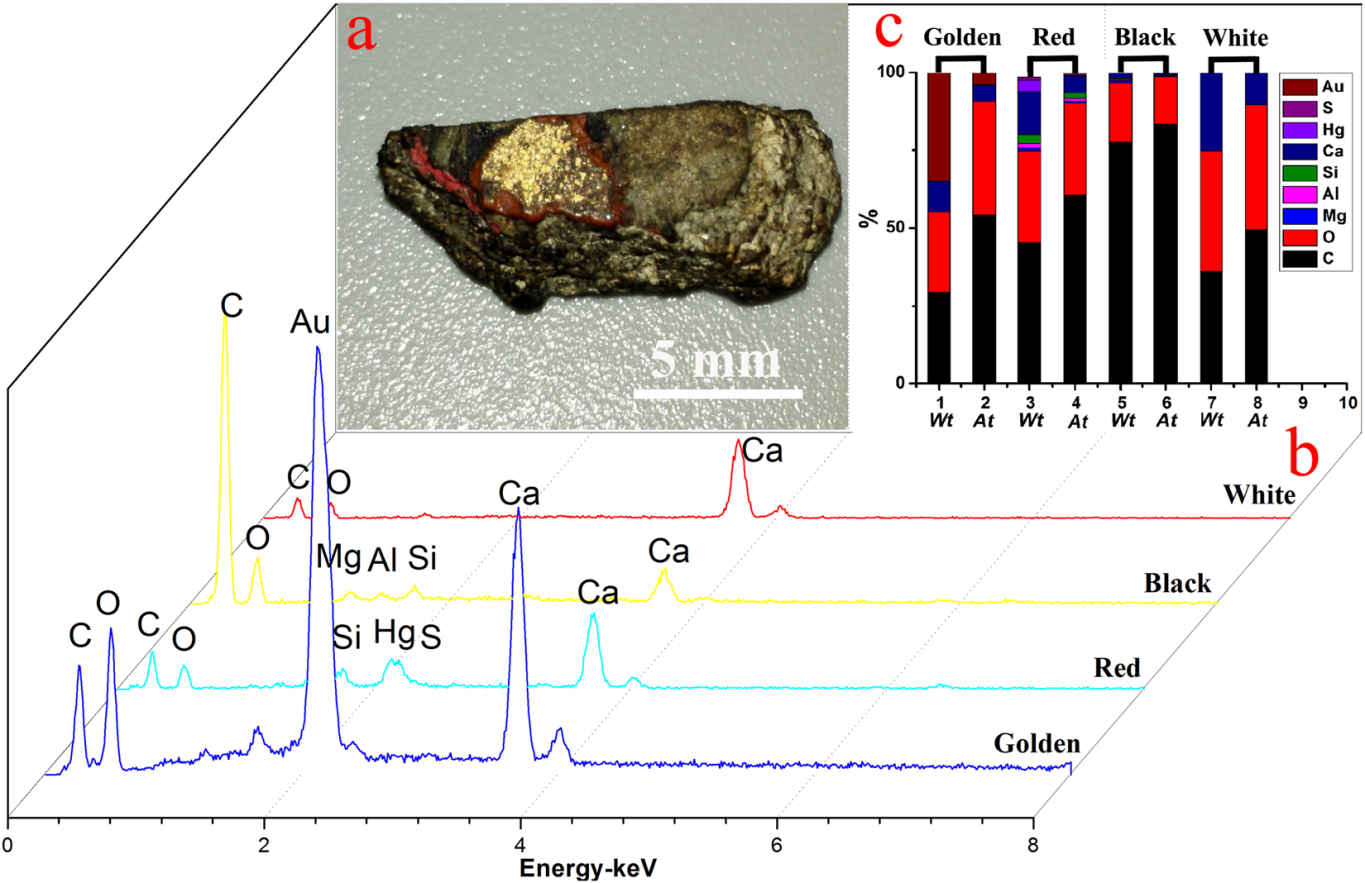
(**a**) Photograph of the sample from the lacquered wooden coffin of Emperor Qianlong, (**b**) EDS results of the four individual pigment layers within its lacquer film pigment layer, (**c**) the corresponding element percentage chart of the four pigment layers.

In order to determine the exact inorganic materials that were used as pigments on the sample, additional EDS tests was applied to analyze the elemental composition of each pigment layer within the lacquer film of the sample (Fig. 2). In Fig. 2a, the uppermost layer is mainly yellow in color, and the main elements were C, O, Au and Ca, with trace elements of Si, suggesting that the surface gilding layer consist of lacquer simply mixed with gold powder. The next layer is in red color and mainly consisted of C, O, Hg, S, Ca and trace amount of Si, with Hg and S as the main contributors to the color, which is likely due to the existence of cinnabar (HgS). The third layer is mainly black in color, and EDS analysis shows large amount of C element and trace amount of O, Mg, Al, Si, Ca, indicating the presence of carbon black. The last layer is a white layer consisted of C, O and Ca, with calcite (CaCO_3_) as the main contributor to the color. These results are consistent with the Raman Spectroscopy analysis results. In conclusion, from the most interior layer to the exterior surface, the lacquer film pigment layer of the sample consists of white calcite (CaCO_3_) layer, black carbon black (C) layer, red cinnabar (HgS) layer and yellow gold (Au) gilding layer. This structure of the lacquer film suggests that the surface of the coffin body might have been repeatedly coated with the lacquer film for a number of times during the manufacturing process. It is also worth mentioning that the element Si exists in each individual pigment layer, suggesting that quartz sand might have been added in the manufacturing process in order to adjust the viscosity of the lacquer sap, and to increase the resistance of the lacquer film against abrasion and moisture, thus improving the quality of the lacquer products.

### The Cross-section Morphology and Composition Analysis

**Figure 3:**
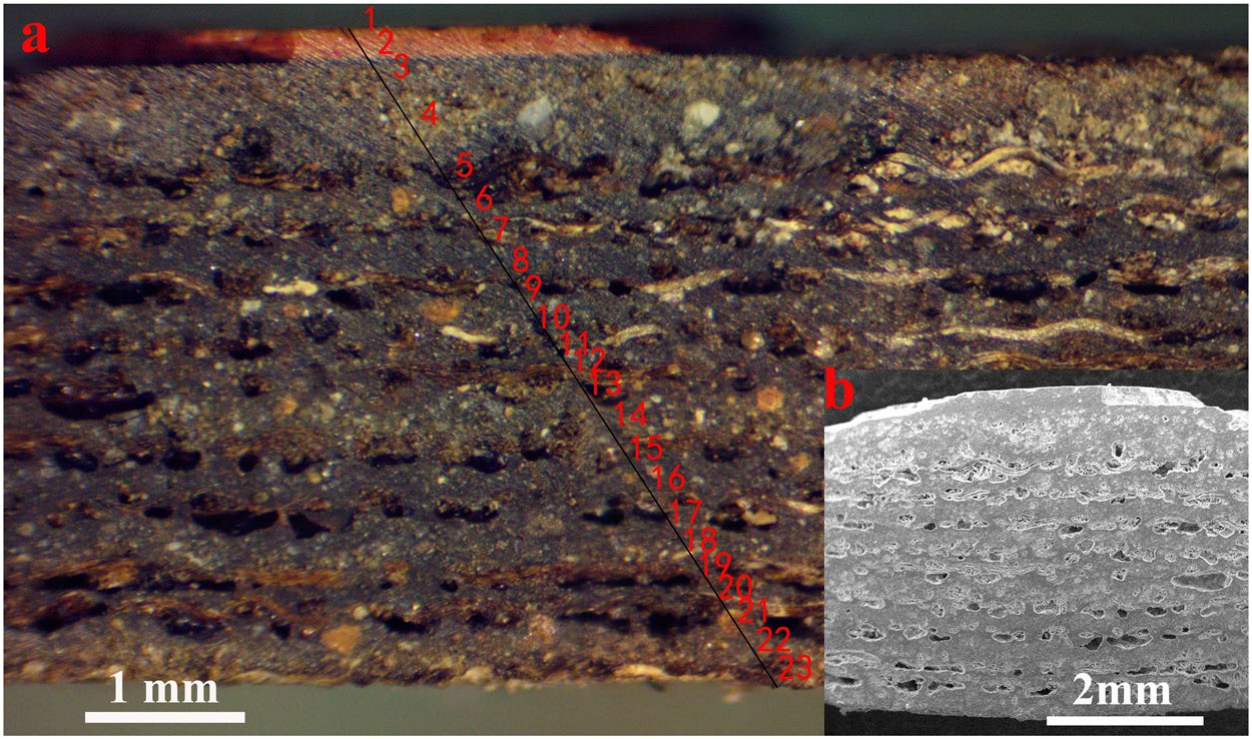
The cross-sectional images of the sample from the lacquered wooden coffin of Emperor Qianlong: (**a**) Stereo Microscope photograph and (**b**) SEM image.

Cross-sectional analysis not only reveals the layered structure of the coffin body, but also shows the appearance of different materials used in the composition. Certain lacquering technique can also be observed from the cross-section image. Figure 3a shows a microscopic photograph of the cross-section of the sample, and Fig. 3b shows its corresponding cross-section SEM image. EDS tests were applied to determine the number of layers within the structure and the elemental composition of each layer. The results show that the sample from the coffin body consists of twenty-three layers in total. From the interior surface to the exterior surface, the coffin body primarily consists of a wooden body layer, a lacquer ash layer and a lacquer film pigment layer.

**Figure 4:**
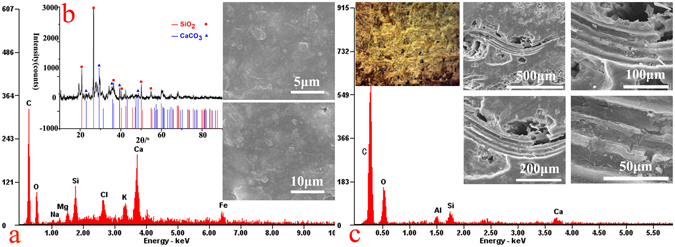
The lacquer ash layer of the sample: (**a**) SEM-EDS of the stucco layer, (**b**) XRD of the stucco layer, (**c**) SEM-EDS of the fiber layer.

The lacquer film pigment layer includes four layers, namely a white calcite (CaCO_3_) layer, a black carbon black (C) layer, a red cinnabar (HgS) layer and a yellow gold (Au) gilding layer. This result is consistent with the surface analysis results. The lacquer ash layer includes nine stucco layers and ten fiber layers on top of each other in an alternating order, providing more evidence for the inference that the coffin surface was lacquered repeatedly during the manufacturing process. The results of SEM-EDS (Fig. 4a) showed that the stucco layer mainly contains C, O, Mg, Al, Si and Ca, which are common elements from clay minerals and calcite. XRD (Fig. 4b) result shows that the stucco layer contains SiO_2_ and CaCO_3_, which were very likely sourced from shattered and grounded porcelain. It was also common practice in that period to source the CaCO_3_ from natural seashells. The purpose of the fiber layers in addition to the stucco layers was likely to increase the durability of the coffin body. In the traditional lacquering craftsmanship, fine silk, cotton or ramie fabrics were commonly pasted on the surface of the wooden body to strengthen it before the lacquering process^3^. Figure 4c shows that the fiber layer predominantly contains C, O and trace amounts of Al, Si and Ca. Warping is observed particularly in the fiber layer, which is characteristic of cotton fibers. The diameters of the fiber are around 15–20 um, which is also consistent with the property of cotton fibers^23–26^. The durable lacquer ash consisted of layers of stucco and fibers also serves the purpose of providing the wooden body underneath extra resistance against abrasion and moisture, in addition to the lacquer film on the exterior surface.

**Figure 5:**
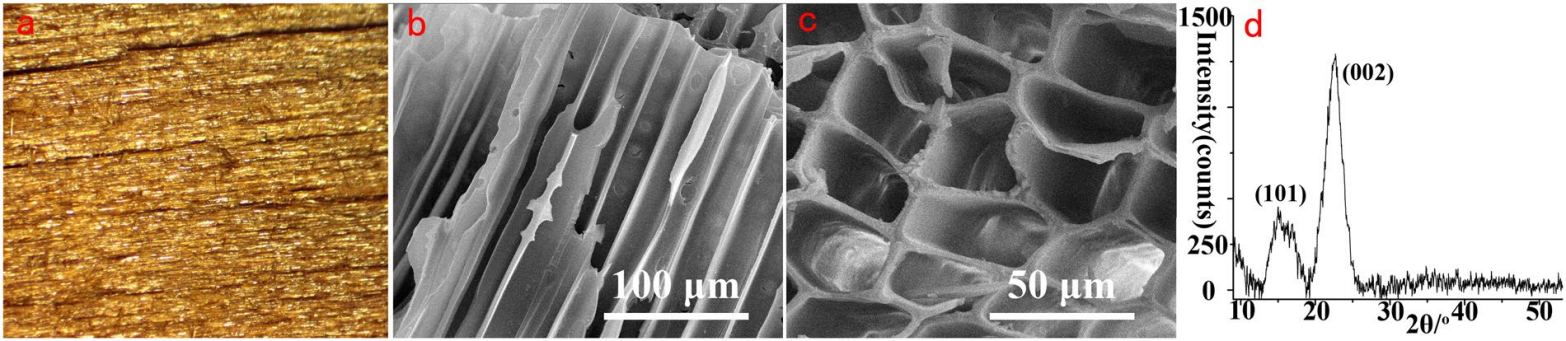
The wooden body layer of the sample: (**a**) Stereo Microscope photograph, (**b**,**c**) SEM images, (**d**) XRD result.

Figure 5a shows the Stereo Microscope photograph of the wooden body layer of the sample, and Fig. 5b,c shows its corresponding SEM images. The cellular structure of the wood remained relatively stable. The crystal structure of cellulose crystallites was examined by XRD, and the result is shown in Fig. 5d. The lower angle peak was the result of the merging of the diffraction peaks at 2θ = 15° and 16.9° into a broader one, where it is assigned to the [101] crystalline plane. The peak observed at 2θ = 22.5° was assigned to the [002] crystalline plane^27–31^. Those results indicate that the wooden body was better preserved, possibly due to the fact that it was covered and protected by the lacquer ash layer and the lacquer film pigment layer on the top.

### Further Analysis on the Lacquer Film Matrix

**Figure 6:**
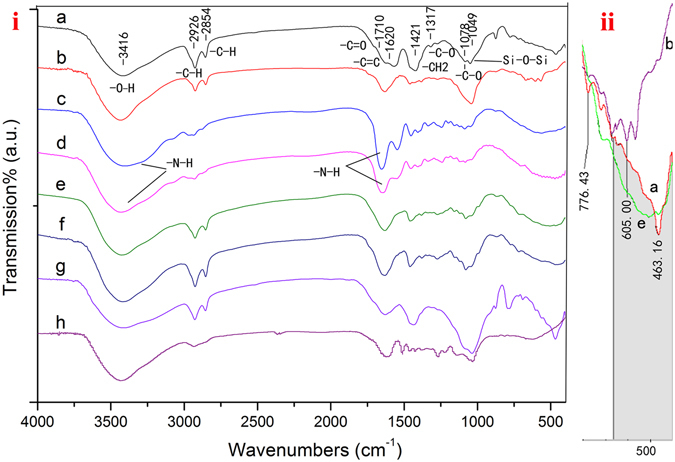
(**i**) On the left (a) The lacquer film sample from the coffin, (b) Fresh modern lacquer film, (c) Cattle bone gelatin, (d) Fish gelatin, (e) Fresh modern lacquer film added with Cattle bone gelatin, (f) Fresh modern lacquer film added with Fish gelatin, (g) The fiber sample from the coffin, (h) The wooden body sample from the coffin. (**ii**) On the right is the enlarged overlay of (a) (b) and (e).

In order to analyze the complex composition of the lacquer film matrix of the coffin, a series of FTIR analysis was carried out to test the ancient samples from the coffin, in comparison with fresh modern lacquer film samples and fresh common organic additive samples. The FTIR spectra of the lacquer film pigment layer sample, the fiber layer sample and the wooden body layer sample from the coffin were shown in Fig. 6i(a,g,h). The spectra of the reference samples were also shown in Fig. 6i(b–f), including a fresh modern lacquer film sample, a cattle bone gelatin sample, a fish gelatin sample, a fresh modern lacquer film added with cattle bone gelatin and a fresh modern lacquer film added with fish gelatin. The spectrum of the ancient lacquer film sample (Fig. 6i(a) shows a specific peak at 3416 cm^−1^ due to O-H stretching and N-H stretching, a peak at 2926 and 2854 cm^−1^ due to C-H stretching, a peak at approximately 1710 cm^−1^ due to the carbonyl group vibration, a peak at 1620 cm^−1^ due to the C=C stretching vibration of the polymerized catechol derivatives, and a peak at 1421 cm^−1^ due to the absorption of C-H bending vibration in a side chain. The absorptions in the range of 1320-1050 cm^−1^, including 1317 cm^−1^ and 1078 cm^−1^, are assigned to C-O stretching in phenol groups and lacquer polysaccharides, respectively^32–34^. The peak at 1049 cm^−1^ suggested the existence of the Si-O-Si bond^35^, indicating the existence of the quartz sand in the lacquer film layer, which is consistent with the surface SEM-EDS analysis results. The shape of the FTIR spectrum of the ancient fiber sample (Fig. 6i(g) was similar to that of the ancient lacquer film sample, which indicates that the lacquer sap was used as a binder within lacquer ash layer. Figure 6ii is the enlarged overlay of FTIR spectra Fig. 6i(a,b and e). The absorptions in the region 550-400 cm^−1^ are due to N-C=S or S-S stretching. It shows that the shape of the FTIR spectra of the ancient lacquer film sample is most similar to the fresh modern lacquer film sample added with cattle bone gelatin. However, the overlay shows significant difference from the fresh modern lacquer film sample without the cattle bone gelatin being added. Based on these results, it is safe to conclude that animal gelatin was added to the lacquer sap during the manufacturing process, in order to disperse the urushiol molecules, to protect and polish the surface of the lacquer, thus improving the quality of lacquer product.

**Figure 7:**
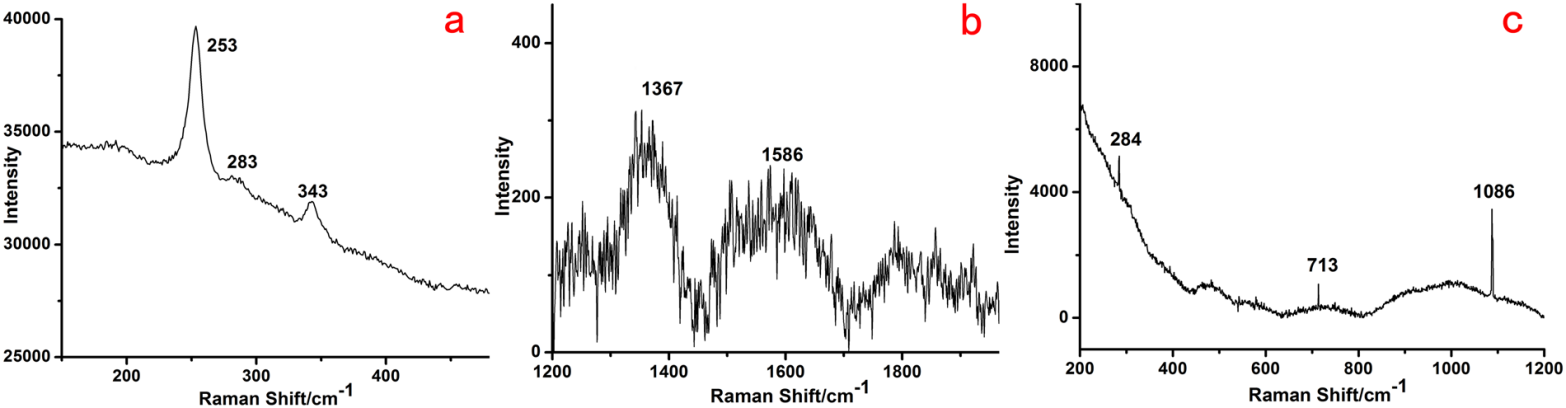
Raman spectra of the lacquered wooden coffin sample of Emperor Qianlong: (**a**) the red pigment layer, (**b**) the black pigment layer, (**c**) the white pigment layer.

The Raman analysis further clarified the composition of the pigments used in the lacquer film pigment layer of the coffin. Cinnabar (HgS) and minium (Pb_3_O_4_) were common red pigments in ancient Chinese artifacts^36–38^. The Raman spectrum of the red pigment was reported in Fig. 7a, showing a very strong signature of cinnabar (HgS), the mercury sulfide mineral with a bright red color, with peaks at 253, 283, and 343 cm^−1^. Its strong ability to maintain bright color for thousands of years and its anti-corrosion nature often make it the ideal choice for a red pigment. From the Raman spectrum of the black pigment (Fig. 7b), it can be easily identified as carbon black, due to the characteristic broad Raman bands at 1367 and 1586 cm^−1^ featuring amorphous carbon^39, 40^. It is usually produced by incomplete combustion of organic matter, such as plants, oil, bones, etc., and has been used as a black pigment from the Paleolithic Age. Figure 7c shows the Raman spectrum of the white pigment. The characteristic bands at 284, 713 and 1086 cm^−1^ reveal that the nature of the white pigment is calcite^14^. Calcite traditionally came from slaked lime, and was widely used as both a white pigment and as a supporting layer in ancient China.

**Figure 8:**
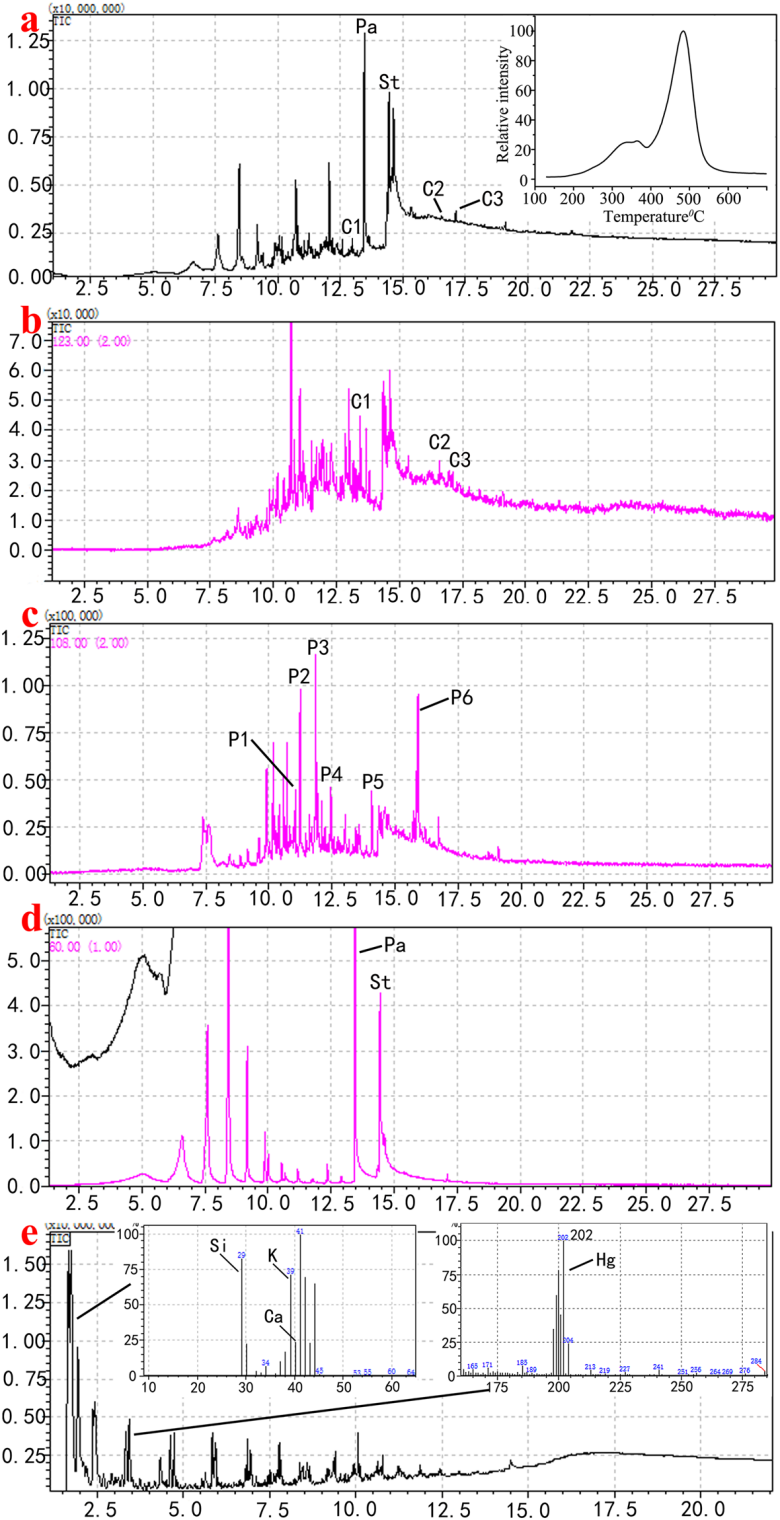
Pyrograms of the lacquered wooden coffin sample of Emperor Qianlong obtained by double-shot Py-GC/MS: (**a**) total ion pyrogram and EGA (Evolved gas analysis) charts; (**b**) m/z 123 extracted ion pyrogram; (**c**) m/z 108 extracted ion pyrogram; (**d**) m/z 60 extracted ion pyrogram; (**e**) total ion pyrogram and mass spectrometry of Si, K, Ca and Hg elements, respectively. Note: C = catechols (C1 = 3-heptylcatechol (MW 318), C2 = 3-pentadecenylcatechol (MW 320), C3 = 3-pentadecylcatechol); P = phenols (P1 = 3-pentylphenol, P2 = 3-hexylphenol, P3 = 3-heptylphenol, P4 = 3-octylphenol, P5 = 3-nonylphenol, P6 = 3-pentadecylphenol); Pa = palmitic acid, St = stearic acid.

**Figure 9:**
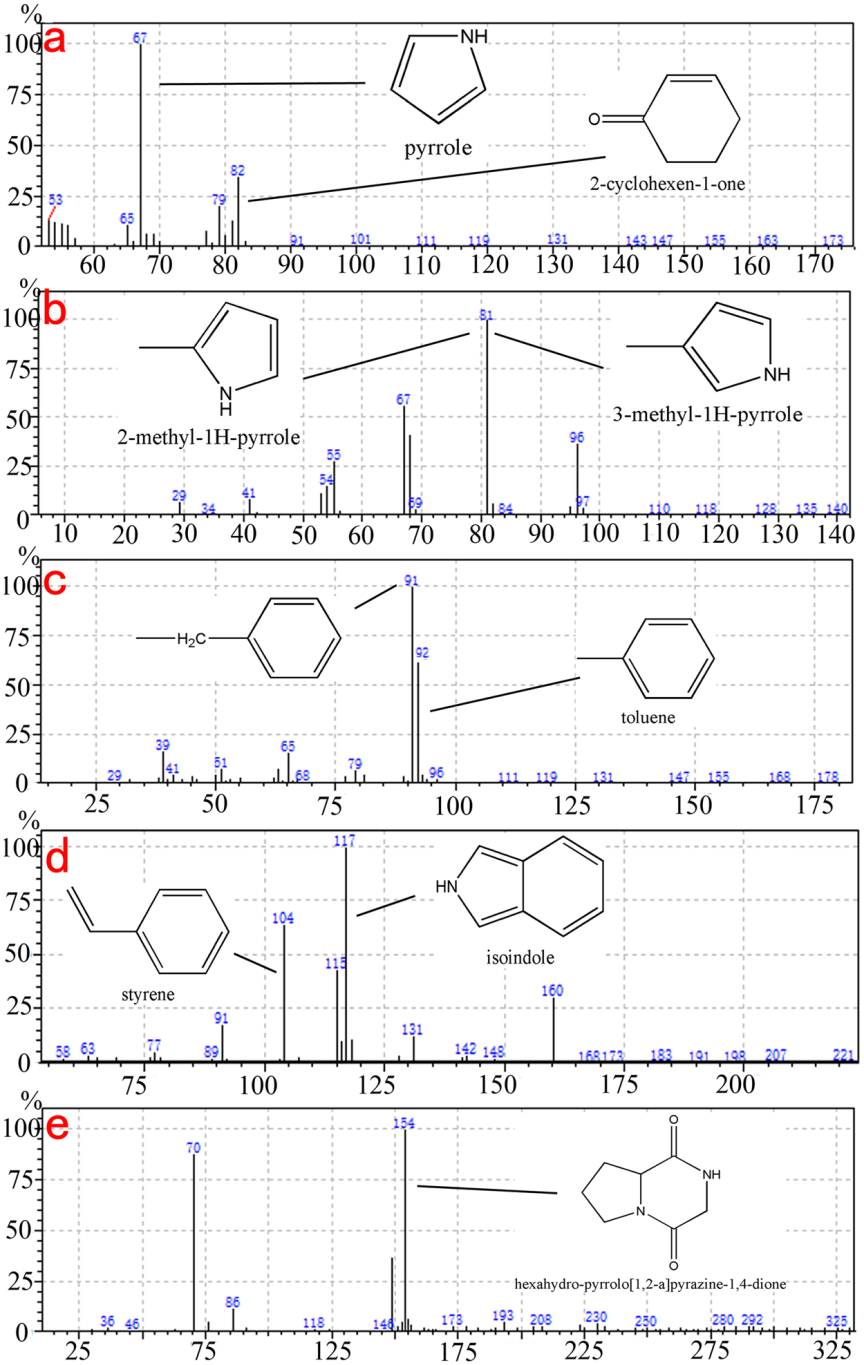
The mass spectra of (**a**) pyrrole (m/z 67) and 2-cyclohexen-1-one (m/z 82); (**b**) 2-methyl-1H-pyrrole (m/z 81) and 3-methyl-1H-pyrrole (m/z 81); (**c**) toluene (m/z 92); (**d**) styrene (m/z 104) and isoindole (m/z 117); (**e**) hexahydro-pyrrolo[1,2-a]pyrazine-1,4-dione (m/z 154).

The additives mixed into the lacquer sap not only protect and polish the lacquer surface, but also improve the process of coloring the lacquer with various pigments. One or more binding media, such as drying oil, animal gelatin, or some inorganic minerals, are usually added to the lacquer film and the lacquer ash. Previous FTIR results informed us the likely existence of such animal gelatin in the lacquer film pigment layer of the ancient sample. In order to gain further information on the full complexity of the lacquer film matrix and to verify the results of previous tests, Double-shot Py-GC/MS analysis was performed, and found to be one of the best methods to identify the chemical composition of the lacquer sap and these additives. The typical pyrolysis products of urushi lacquer (Rhus. Vernicifera) are the urushiol constituents, among which 3-pentadecenylcatechol and 3-pentadecylcatechol were detected in the sample (Fig. 8a)^36^. Aside from the pentadecylcatechols that have the longest side chains among the catechols, a peak assigned to 3-heptylcatechol was also identified (Fig. 8a). By using extracted ion technique, the alkylcatechols (m/z 123) and alkylphenols (m/z 108) were detected by their base peaks, which are shown in the extracted ion pyrograms in Fig. 8b,c. The highest relative intensity was observed for 3-heptylphenol, which is also a characteristic pyrolysis constituent of the Rhus vernicifera. The presence of the three distinctive catechols along with the other catechols’ and phenols’ profiles based on the m/z 123 and m/z 108 ions clearly indicate a good correspondence of the constituents with the reference standard of urushi lacquer (Rhus. Vernicifera)^1, 41, 42^. Figure 8d shows that high amounts of short-chain fatty acids, palmitic acid and stearic acid were detected, indicating that a drying oil was added to the lacquer film matrix. The function of the drying oil might have been used as a solvent to decelerate the hardening process of the urushi, and to increase its lustre and elasticity^43, 44^. For the elemental analysis result of the Double-shot Py-GC/MS, Si, K, Ca elements were detected (Fig. 8e). In addition, Hg element was identified with the most abundant isotopes of the element: m/z 199, 200, 202 (Fig. 8e)^45, 46^. This result suggests that calcite (CaCO_3_) is most likely the white pigment in the lacquer film, and cinnabar (HgS) the red pigment. The existence of the element Si also indicates that the quartz sand (SiO_2_) was added to the lacquer film matrix. These results verify the earlier conclusions drawn from the previous EDS and Raman analysis. In the meantime, other chemical compounds, including pyrrole, 2-cyclohexen-1-one, 2-methyl-1H-pyrrole, 3-methyl-1H-pyrrole, toluene, styrene, isoindole and hexahydro-pyrrolo[1,2-a]pyrazine-1,4-dione were also identified in the lacquer film matrix (Fig. 9a–e). These compounds are found to be the marker pyrolysis products of the proteins by comparing to the reference proteinaceous materials^47^.

### Investigation on the lacquering techniques on the coffin of Emperor Qianlong

The lacquering craftsmanship on the ancient lacquered wooden coffins reflects an important part of the funerary culture of the Qing dynasty. Based on the analytical results that were discussed, the manufacturing process of the lacquered wooden coffin of Emperor Qianlong is identified as four main stages. Firstly, craftsmen started by producing the wooden body of the coffin. According to the structure of the coffin body that was observed in the cross-section image, the second step was to apply the lacquer ash layer onto the surface of the wooden body. This step was a vital part of the process that determined the final quality of the lacquerware. The application of the lacquer ash can be deduced based on the composition of the lacquer ash layer of the coffin sample, which was identified in the cross-section analysis and the FTIR results described above. The lacquer ash layer consists of nine layers of stucco and ten layers of fiber in an alternating order, with lacquer sap as a binding medium. Therefore, it is safe to conclude that during the application, the fiber layer, likely a paste of cotton fabric immersed in lacquer sap, is first applied onto the wooden surface. Then a paste of mixed stucco and lacquer sap was applied on top of the fiber layer. The stucco was likely grounded into fine particles and added to the lacquer sap to obtain a desired viscosity. This process was applied repeatedly in an alternating manner until the layers were well adhered and the wooden body sufficiently coated, a technique that was referred as the “wan lacquering”. The third step was the application of the lacquer film pigment layer on top of the lacquer ash. Based on the results of the lacquer film matrix analysis, it is deduced that the lacquer film was produced from mineral pigments, lacquer sap, animal gelatin, drying oil, quartz sand (SiO_2_), and proteinaceous materials. Each layer of the lacquer film was prepared from these constituents, mixed with appropriate ratios, colored with different mineral pigments, and then brushed onto the surface. As concluded from the surface composition analysis, a white calcite (CaCO3) layer, a black carbon black (C) layer, a red cinnabar (HgS) layer and a yellow gold (Au) gilding layer were brushed onto the surface several times to form the corresponding colors in the lacquer film. As a finishing step, the surface of the lacquered wooden coffin was polished.

For the application of the lacquer ash layer and the lacquer film pigment layer, several distinctive lacquering techniques were identified on the sample from the coffin. The “wan lacquering” technique can be observed from the lacquer ash layer, which refers to the repeated process of pasting the fiber layer onto the stucco, and then pasting the stucco onto the fabric, with the help from the added lacquer sap. This special technique was found to be able to smooth the lacquer ash layer, and build a solid foundation for the subsequent lacquer film applications. The mixing technique “Jin Jiao” was identified for mixing the pigment into the prepared lacquer film matrix, while another lacquering technique “painting lacquer above the gold” was observed on the gilding layer, which is self-explanatory. These lacquering techniques can not only increase the coffin body’s stability and mechanical strength, but also improve the aesthetic values of the surface decorations on the coffin. For the gilding layer, the coffin of Emperor Qianlong used pure gold while the coffins of the general and his empresses used alloys. Such limitation and regulation on the choices of funerary materials reflect the rigorous hierarchical system of the ancient China in the Qing Dynasty period.

## Conclusion

The composition, structure and lacquering techniques of the lacquered wooden coffin of Emperor Qianlong were systematically investigated using multiple scientific methods. Surface analysis of a sample from the imperial coffin, performed by Stereo Microscope, SEM-EDS Imaging and Mapping, indicates that the surface of the coffin undergoes considerable deterioration and effective conservation measures should be taken to prevent further damage. The cross section image of the sample illustrates the primary structure of the coffin body consists of a wooden body layer, a lacquer ash layer and a lacquer film pigment layer. The lacquer ash layer includes nine stucco layers and ten fiber layers in an alternating order. FTIR results show that the lacquer sap was used as a binder in the stucco layer and fiber layer. Animal gelatin was added to help disperse the urushiol molecules, and to protect and polish the surface of the lacquer. The EDS mapping and Raman analysis clarified the lacquer film pigment layer consists of a white calcite (CaCO_3_) layer, a black carbon black (C) layer, a red cinnabar (HgS) layer and a yellow gold (Au) gilding layer, indicating that the lacquer film was applied by a number of repeated brushing applications. Based on the combined results of SEM-EDS Mapping, FTIR, Raman and Double-shot Py-GC/MS, it is concluded that the composition of the lacquer film matrix includes mineral pigments, lacquer sap, animal gelatin, drying oil, quartz sand and proteinaceous materials. The presence of the three distinctive catechols along with the other catechols’ and phenols’ profiles based on the m/z 123 and m/z 108 ions clearly indicate the species of the lacquer tree was Rhus vernicifera. The lacquering techniques “wan lacquering”, “painting lacquer above the gold” and “Jin Jiao” were identified as part of the investigation on the manufacturing process of the coffin. This investigation provides scientific evidence that helps archaeologists and historians in the field to gain further understanding on the imperial lacquering craftsmanship and funerary culture in the Qing Dynasty. This project also establishes a foundation of technical studies on the current conditions of the coffin for future preservation and conservation purposes.

## Methods

The lacquered wooden coffin (Outer Coffin) sample of Emperor Qianlong was collected from the Eastern Imperial Tombs of the Qing Dynasty, Tangshan, Hebei province and is available for this study (Provided by Jingzhou Preservation Centre of Cultural Relics). The reference modern lacquer film samples with organic additives (sample b-f) were fabricated from fresh lacquer sap extracted from certified Rhus vernicifera. The fresh Rhus vernicifera lacquer sap was collected from Hubei Province. The lacquer sap was processed by traditional methods and was coated on glass wafers. Sample b was fresh modern lacquer film with no additives. Sample e was fresh modern lacquer film with cattle bone gelatin. Sample f was fresh modern lacquer film with fish gelatin. The lacquer film samples were approximately 80 μm thick and were stored for 7 days in a humidity-controlled chamber at 25–35 °C with 75% relative humidity. The films were then left to air dry for 2 to 4 months.

Stereoscopic Microscopy, SEM-EDS, XRD, FTIR, Raman, Py-GC/MS were used as scientific analytical methods. The detailed information for the instrumental analysis is given in the Supplementary information section.

## Electronic supplementary material

Supplementary information
