# Supplementary material for: Analysis on the Composition/structure and Lacquering Techniques of the Coffin of Emperor Qianlong Excavated from the Eastern Imperial Tombs
Source: Sci Rep. 2017 Aug 24;7:8446. doi: 10.1038/s41598-017-08933-8 (PMC5570907; doi:10.1038/s41598-017-08933-8)
Supplement: Supplementary file 1 — Supplementary information [file 41598_2017_8933_MOESM1_ESM.doc]

**Analysis on the Composition/structure and** **Lacquering Techniques of** **the Coffin of Emperor Qianlong Excavated from** **the**

**Eastern Imperial Tombs**

**Xinying Hao****^a,d^, Hao Wu^b^, Yang Zhao^b^, Tong Tong^c^, Xiaoyuan Li^a^, Cui Yang^a^, Yun Tang^a^, Xinyu Shen^a^, Hua Tong^a,d^,***

^a^ Key Laboratory of Analytical Chemistry for Biology and Medicine, Ministry of Education, College of Chemistry and Molecular Sciences, Wuhan University, Wuhan 430072, China

^b^ Jingzhou Preservation Centre of Cultural Relics, Jingzhou 434020, China

^c^ Centre of Cultural Material Conservation, The University of Melbourne, Parkville, VIC 3010, Australia

^d^ Archaeology Research Center of Science and Technology, Wuhan University, Wuhan 430072, China

* Corresponding author at: Key Laboratory of Analytical Chemistry for Biology and Medicine, Ministry of Education, College of Chemistry and Molecular Sciences, Wuhan University, Wuhan 430072, China. Tel.: +86 2768764510; fax: +86 2768752136. E-mail address: sem@whu.edu.cn (H. Tong).

**Surface and cross-sectional observations**

The surface and cross-sectional observations were conducted using a stereoscopic microscope (XTL-165, Phoenix, China) in order to detect all layers of the sample's constitution. Scanning Electron Microscope (SEM) images of the lacquered wooden coffin samples were obtained with a Quanta 200 Scanning Electron Microscope (FEI, Netherlands) at 30 keV accelerating voltage.

**SEM-EDS**

The instrument applied for SEM-EDS analysis was a SEM (Quanta 200, Holland) coupled with an Energy Dispersive Spectrometer (GENSIS, AMETEK, USA). The accelerating voltage was set at 5 kV and the working distance at 8 mm. Analyses were conducted without evaporative coating and the conducting carbon tape was used to fix the sample to the sample stage.

**FTIR**

The FTIR spectra of all the samples were recorded using a Fourier transform infrared spectroscopy (NICOLET 5700, USA) by adopting the potassium bromide tabletting method. Spectra over the range of 4000-400 cm^-1^ were collected with a resolution of 4 cm^-1^ and 64 scans.

**XRD**

X-Ray Diffraction (XRD) of the stucco samples and wooden body sample were performed using an Xpert powder diffractometer (PANalytical, Netherlands) with Cu Kα radiation from a source operated at 40 kV and 40 mA. Samples were gently ground and analyzed over a diffraction-angle (2*θ*) range of 5-90 ^o^ or 60^o^, at a step size of 0.02 ^o^ and a dwell time of 1 s.

**Raman analysis**

The Raman spectra were recorded on a Raman microspectrometer (LabRam HR800, Jobin Yvon, France). A 785-nm near-infrared (NIR) diode laser was used as the excitation source. Laser focus can be achieved by adjusting the height of the microscope objectives instead of the sample stage. The spectral resolution was～0.8 cm^-1^ and the optical cable line was～600 line/mm.

In order to eliminate the background signal, the different wavelength lasers were used and finally the 785-nm excitation source was chosen. Considering the tiny laser spot and the mixture nature of the lacquer film pigment layer, several measurements had to be made around each acquisition spot to get the signals from pigment layer and exclude the signals from the impurities or contaminants on the surface. The integral time was prolonged to 40 s and 8 acquisitions were averaged in order to improve the spectral signal-noise ratio.

**Double-shot Py-GC/MS**

For the double-shot Py-GC/MS analysis about 0.3 mg of sample was placed in a sample cup. The cup was placed on top of the pyrolyzer at near ambient temperature and then introduced into the furnace by the autosampler, afterwards the temperature program of the GC/MS was started.

The pyrolysis-gas chromatography/mass spectrometry measurements were carried out using a multi-shot pyrolyzer EGA/PY-3030D (Shimadzu,Japan) and a GCMS-QP2010 Ultra (Shimadzu, Japan). A stainless steel capillary column (0.25 mm i.d. × 30 m) coated with 0.25 mm of Ultra Alloy PY-1 (100% methylsilicone) was chosen in order to provide an adequate separation of the components. The chromatographic conditions were as follows: the oven initial temperature was 40 ^0^C with a gradient of 20 ^0^C min^-1^ to 320 ^0^C, which was held for 14 min, the second step pyrolysis was set at 600 ^0^C. The carrier gas was Helium with an inlet pressure of 20 kPa and 1:100 split ratio. The electronic pressure control was set to the constant flow mode. Ions were generated by electron ionization (70 eV) in the ionisation chamber of the mass spectrometer. The mass spectrometer was scanned from m/z 30 to 800 with a cycle time of 0.5 s. EI mass spectra were acquired by total ion monitoring mode. The temperatures of the interface and the source were 280 and 230 ^0^C, respectively.
